# Prometastatic Potential of Non-Functionalized Multiwalled Carbon Nanotubes in the MDA-MB-436 Breast Cancer Cell Line Model

**DOI:** 10.3390/ijms26062777

**Published:** 2025-03-19

**Authors:** Magdalena Matysiak-Kucharek, Krzysztof Sawicki, Marcin Kruszewski, Jacek Kurzepa, Lucyna Kapka-Skrzypczak

**Affiliations:** 1 Department of Molecular Biology and Translational Research, Institute of Rural Health, 20-090 Lublin, Poland; matysiak.magdalena@imw.lublin.pl (M.M.-K.); sawicki.krzysztof@imw.lublin.pl (K.S.); 2 Center for Radiobiology and Biological Dosimetry, Institute of Nuclear Chemistry and Technology, 03-195 Warsaw, Poland; m.kruszewski@ichtj.waw.pl; 3 Department of Medical Chemistry, Medical University of Lublin, 20-093 Lublin, Poland; jacek.kurzepa@umlub.pl; 4 World Institute for Family Health, Calisia University, 62-800 Kalisz, Poland

**Keywords:** MDA-MB-436, breast cancer, multiwall carbon nanotubes, cytotoxicity, oxidative stress, inflammation, apoptosis, metastasis, epithelial–mesenchymal transition

## Abstract

Multiwalled carbon nanotubes (MWCNTs) are used in many areas of industry and medicine. However, there is evidence suggesting profibrogenic action of MWCNTs, probably via the epithelial–mesenchymal transition mechanism (EMT). The aim of this study was to evaluate the prometastatic activity of 5–20 nm and 50–80 nm MWCNTs against cells of the MDA-MB-436 line. We used MTT and NR assays to determine MWCNTs’ cytotoxicity and the level of malonylodialdehyde and thiol compounds as indicators of oxidative stress. qRT-PCR was used to examine the expression of EMT markers. The QCM Chemotaxis Cell Migration Assay was used to assess cell migration, while the Cytokine Array Kit and Apoptosis Array Kit were used to determine cytokine expression and induction of apoptosis. The interleukin 6, C-X-C motif chemokine ligand 8, and tumor growth factor beta 1 (TGFB1) secretion was determined by ELISA. MWCNTs were toxic to MDA-MB-436 cells and induced cell death via the apoptosis pathway. MWCNTs induced a low level of oxidative stress and were associated with increased secretion of pro-inflammatory cytokines and chemokines, including proteins important in breast cancer metastasis. Cells incubated with MWCNTs showed increased expression of mesenchymal EMT markers. However, in contrast to these results, the migration of MWCNT-treated cells increased only modestly relative to untreated cells. Also, the secretion of TGFB1, a key inducer and regulator of EMT, increased only slightly. In summary, the multifaceted effect of MWCNTs on cancer cells encourages further work on the safety of nanomaterials.

## 1. Introduction

Carbon nanotubes (CNTs) are an allotropic version of carbon, created by folding a single-atom graphite plane, which makes them resemble a hollow cylinder. They are a one-dimensional nanomaterial. The basic technique for obtaining them is the slow condensation of hot vapors of carbon atoms. A characteristic feature of CNTs are excellent mechanical properties—they have very good tensile strength, flexibility, and elasticity. CNTs also have unique electrical properties. Depending on the parameters of diameter and twist degree, they exhibit the properties of conductors or semiconductors. Moreover, they are characterized by resistance to high temperatures and have good thermal conductivity. They also have strong absorption properties, are diamagnetic, and have good chemical properties. Due to the above-mentioned properties, CNTs have been used in many areas of the economy. Pristine, unfunctionalized CNTs are used, e.g., in nanoelectronics and nanoengineering, e.g., as molecular transistors, field emitters, and STM tips [1].

Recent research indicates a broad spectrum of toxic effects of CNTs. One of the best explained mechanisms of their toxicity is oxidative stress induction, which may result in cell cycle disruption, geno- and cytotoxicity, and consequently, mutagenicity and carcinogenicity. CNTs absorbed into the body through the respiratory tract can translocate to other tissues and organs and potentially cause systemic effects, such as neurotoxicity or toxicity of the cardiovascular system. Studies have also shown immunomodulatory effects of CNTs, which leads to immune system dysregulation [2,3,4]. A documented adverse effect of CNTs is also the secretion of increased amounts of inflammatory mediators and pulmonary fibrosis that, as proposed by some authors, may occur through an epithelial–mesenchymal transition (EMT) mechanism [5,6]. This transition is a process involving a cascade of events that leads to acquisition of mesenchymal phenotype by epithelial cells. Epithelial cells are characterized by apico-basal polarization, strong intercellular adhesion, and limited migration potential. Strong intercellular adhesion is the result of several types of junctions, e.g., tight junctions, desmosomes, adhesion zones, and gap junctions. During EMT, epithelial cells acquire a mesenchymal phenotype, and their morphology becomes similar to that of fibroblasts (spindle shape). Apico-basal polarization is replaced by anteroposterior polarization. Tight intercellular junctions are replaced by cell–cell contact junctions and integrin-dependent adhesion to the extracellular matrix. Cells after EMT are characterized by increased motility and invasiveness. EMT occurs in physiological conditions, but also in pathological situations, such as organ fibrosis (EMT leads to the transformation of cells of non-mesenchymal origin, becoming a source of myofibroblasts, mainly responsible for the synthesis of extracellular matrix components, which play a central role in the pathophysiology of fibrosis) or cancer metastasis. Several reports suggest that carbon-based and other nanomaterials (NMs) can modulate EMT in cancer cells in vitro [7,8,9,10,11,12,13,14,15].

Triple-negative breast cancer (TNBC) is characterized by an aggressive course: rapid growth and low histological differentiation of the primary tumor, high risk of distant metastases, poor response to treatment, and early relapse after treatment. It has been shown that in breast cancers, cells in hybrid EMT stages are responsible for lung metastasis, while cells that have undergone a full transformation and reached a mesenchymal state are responsible for increased resistance to chemotherapy treatment [16,17].

Considering the current state of knowledge on this topic, this study aimed to assess the prometastatic potential of multi-wall CNTs (MWCNTs) in human MDA-MB-436 breast cancer cells. We examined the effect of MWCNTs on MDA-MB-436 cell viability and the level of oxidative stress. Then, we determined the effect of MWCNTs on the expression of a number of EMT markers, as well as the level of cell migration and the secretion of TGFB1. Finally, we tested the effect of MWNTs on secretion of proinflammatory cytokines and apoptosis markers.

## 2. Results

### 2.1. Effect of MWCNTs on Viability of MDA-MB-436 Cells

The cytotoxicity of MWCNTs against MDA-MB-436 cells was measured by two methods, namely MTT and Neutral Red assays. To eliminate possible interference of MWCNTs with the assays, we chose assays based on different principles. MWCNT treatment caused a statistically significant reduction in MDA-MB-436 cell viability when measured by MTT assay. For 5–20 nm MWCNTs, a plateau in toxicity was observed after 24 h for 25–100 ug/mL concentrations. After 48 h incubation and for 50–80 nm MWCNTs the trends were similar, although less pronounced (Table 1). Cytotoxicity measured by the NR assay was smaller than that measured by MTT assay. The 5–20 nm MWCNTs were clearly toxic at a concentration of 100 ug/mL, and the 50–80 nm MWCNTs only after 48 h (Table 2). Since MWCNT cytotoxicity was relatively low only in the case of 100 μg/mL 50–80 nm MWCNTs measured with the MTT test after 48 h of incubation, which dropped slightly below 50% survival, the IC50 value was not determined, as it might not reflect real values. Concentrations of 10 µg/mL and 50 µg/mL were selected for further testing.

### 2.2. Effect of MWNCTs on Oxidative Stress Markers

**Figure 1:**
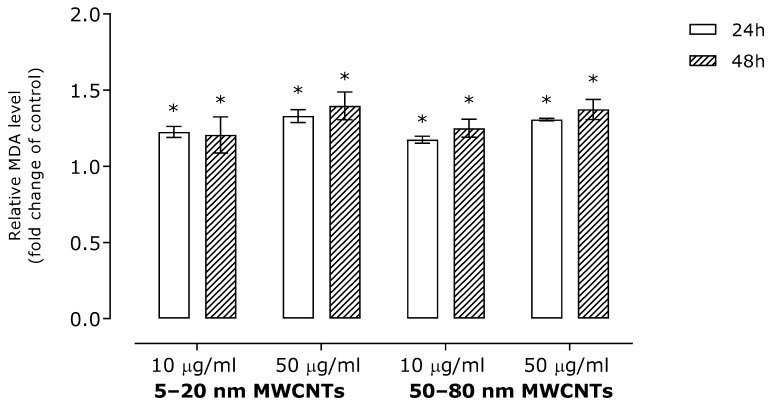
Relative malonyldialdehyde level (MDA) in MDA-MB-436 cells treated with MWCNTs. The presented graph shows the fold change of MDA in samples incubated with MWCNTs as compared with the untreated control. The data were expressed as a mean ± SD (*n* = 3). The asterisk denotes statistical significance at *p* < 0.05.

**Figure 2:**
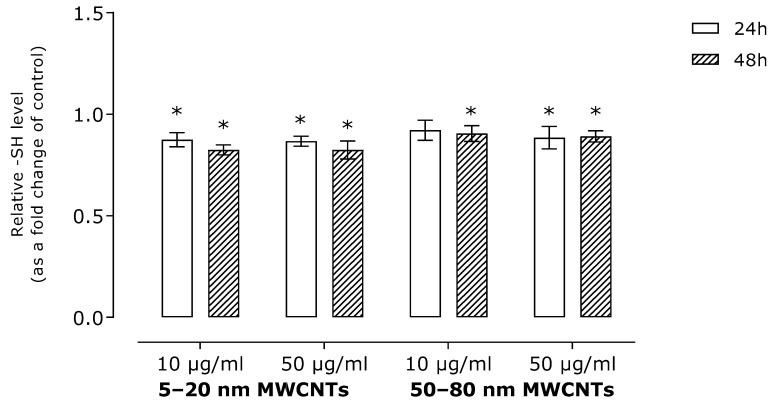
The level of free -SH groups in MDA-MB-436 cells treated with MWCNTs presented as a fold change of free -SH groups in samples incubated with MWCNTs in comparison to the untreated control. The data were expressed as mean ± SD (*n* = 3). The asterisk denotes statistical significance at *p* < 0.05.

To assess the impact of MWCNTs on oxidative stress induction, we determined the level of malonyldialdehyde (a lipid peroxidation marker) and free -SH groups (a marker of global oxidative stress). The results revealed a modest, although statistically significant, increase in MDA level (Figure 1). In line with this, MWCNTs slightly reduced the number of free -SH groups (Figure 2).

### 2.3. Effect of MWCNTs on Expression of Markers EMT, Migration, and TGFB1 Secretion

**Figure 3:**
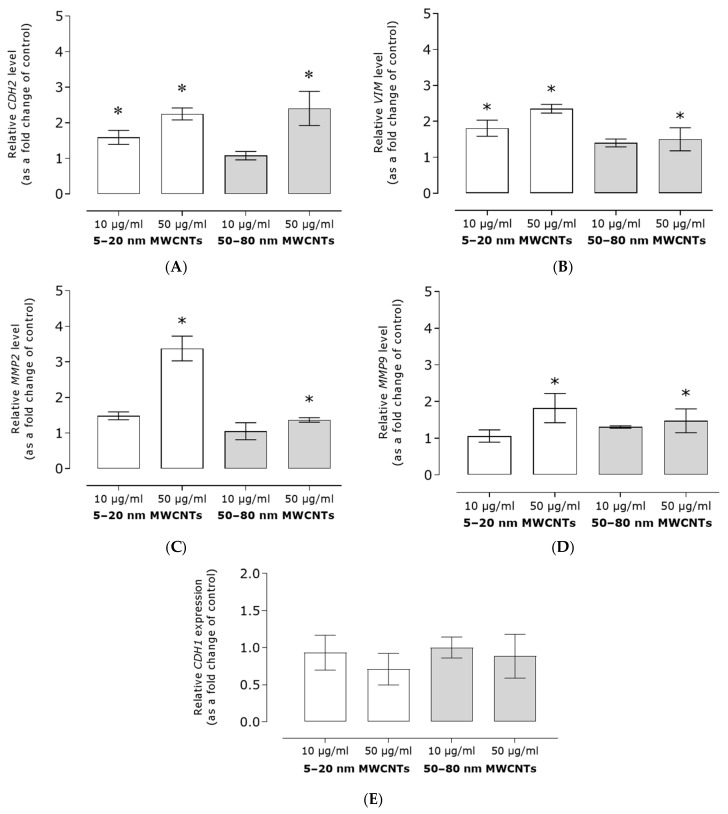
Expression of EMT markers: CDH2 (**A**), VIM (**B**), MMP2 (**C**), MMP9 (**D**), and CDH1 (**E**) in MDA-MB-436 cells treated with MWCNTs. The graph shows the fold change in the level of the tested gene calculated for samples incubated with MWCNTs in relation to the untreated control. β-actin was used as a reference gene. Mean ± SD (*n* = 3). The asterisk denotes statistical significance at *p* < 0.05.

Analysis of the level of mRNA of EMT markers revealed that MWCNTs increased expression of all tested markers. The relative mRNA expression of *CDH2*, *VIM*, and *MMP9* increased approximately twofold, whereas *MMP2* mRNA expression increased threefold as compared to the untreated control. Furthermore, smaller MWCNTs had a much stronger effect on *MMP2* and *VIM* expression than their larger counterparts (Figure 3A–D). The studies showed also that MDA-MB-436 cells were characterized by a relatively low expression of *CDH1*, as indicated by the high Ct values. The expression of this marker was decreased by MWCNT treatment; however, due to high standard deviations, the effect was not statistically significant (Figure 3E).

**Figure 4:**
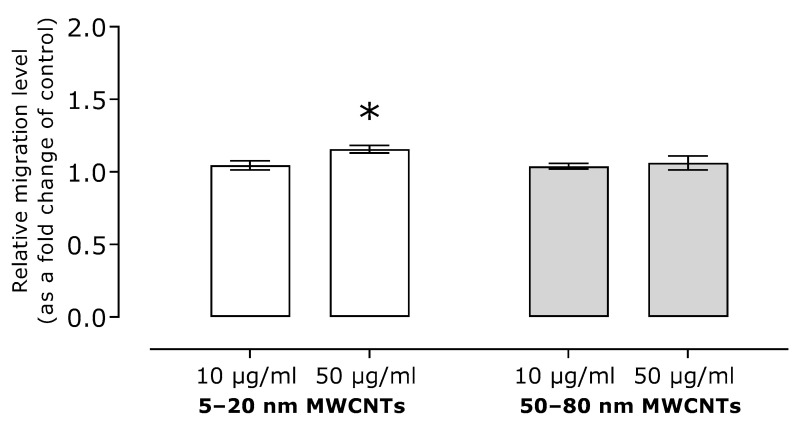
Migration of MDA-MB-436 cells treated with MWCNTs for 24 h. The graph shows the fold change in the migration calculated for samples incubated with MWCNTs relative to the untreated control. Mean ± SD (*n* = 3). The asterisk denotes statistical significance at *p* < 0.05.

Our research showed also that migration of MDA-MB-436 cells treated with MWCNT, measured using a Boyden chamber, was similar that of the control cells. A statistically significant increase in migration was observed only for the higher concentration of 5–20 nm MWCNTs; however, migration was only 1.15 times higher than that of the control cells (Figure 4).

**Figure 5:**
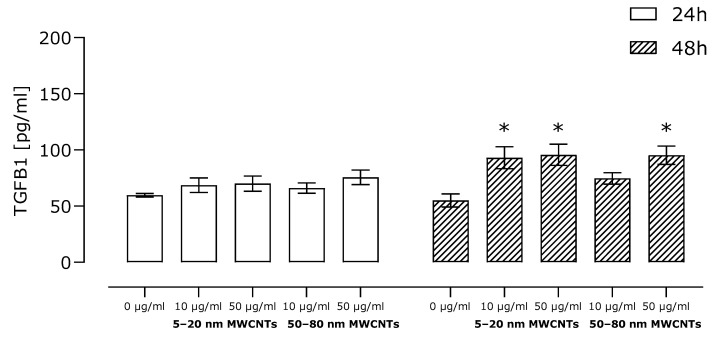
Relative level of TGFB1 secretion by MDA-MB-436 cells treated with MWCNTs for 48 h. Fold change of protein level calculated for samples incubated with MWCNTs as compared to the untreated control. Mean ± SD (*n* = 3). The asterisk denotes statistical significance at *p* < 0.05.

A key factor responsible for the stimulation of EMT in cancer cells is TGFB1; thus, we determined its secretion using the ELISA technique. The results revealed a small, although statistically significant, increase in secretion of this protein in cells incubated with MWCNTs compared to the untreated control (Figure 5).

### 2.4. Effect of MWCNTs on Secretion of Pro-Inflammatory Proteins

**Figure 6:**
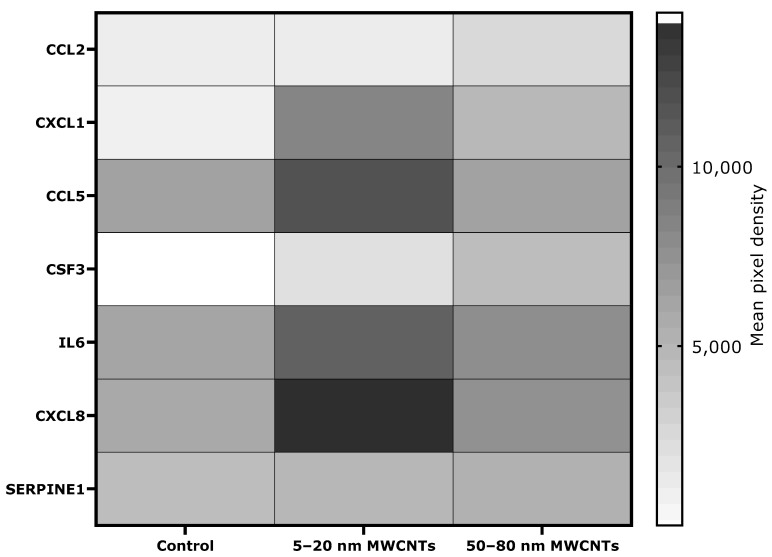
Pro-inflammatory proteins secreted by MDA-MB-436 cells treated with 50 µg/mL MWCNTs for 48 h. The semi-quantitative Proteome Profiler Human Cytokine Array Kit was used in a mixture of cell mediums from three independent experiments. Protein levels are presented as a mean spot pixel density of two individual measurements, normalized to the reference spots and subtracted for background control.

The Human Profiler Cytokine Array (R&D Systems, Minneapolis, MN, USA) was used to access secretion of pro-inflammatory proteins. The array allows for a simultaneous determination of 36 proteins related to the inflammatory process (Supplementary Materials Table S1). Using the profiler, we were able to demonstrate secretion of seven proinflammatory proteins by MDA-MB-436 cells treated with MWCNTs. The analysis revealed that MWCNTs changed the secretion level of six of them: CC motif chemokine ligand 2 (CCL2), chemokine (CC motif) ligand 1 (CXCL1), chemokine (CC motif) ligand 5 (CXCL5), interleukin 6 (IL6), C-X-C motif chemokine ligand 8 (CXCL8) (previously known as interleukin 8), and serpin family E member 1 (SERPINE1). In addition, colony-stimulating factor 3 (CSF3) was detected in the supernatant of MWCNT-treated MDA-MB-436 cells, whereas it was absent in the control cells (Figure 6).

**Figure 7:**
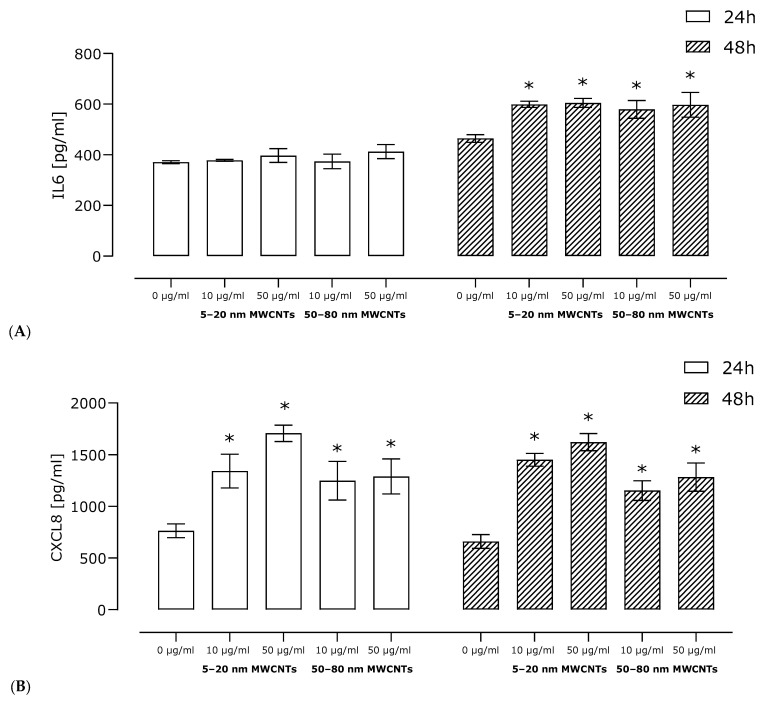
Relative levels of IL6 (**A**) and CXCL8 (**B**) secreted by MDA-MB-436 cells treated with MWCNTs for 24 or 48 h. The graph shows the fold change in protein level calculated for samples incubated with MWCNTs in relation to the untreated control. Data are expressed as mean ± standard deviation (*n* = 3). The asterisk denotes statistical significance at *p* < 0.05.

For two pro-inflammatory proteins crucial in breast cancer metastasis and EMT process, IL6 and CXCL8, semi-quantitative results of the Human Profiler Cytokine Array Kit were verified by ELISA in a wider range of concentrations and incubation times. ELISA studies confirmed the results obtained in the profiler, indicating that both 5–20 nm MWCNTs and 50–80 nm MWCNTs increased secretion of both cytokines. Shorter MWCNTs were more potent inductors of the cytokines (Figure 7).

### 2.5. Effect of MWCNTs on Apoptosis Markers in MDA-MB-436-Treated Cells

**Figure 8:**
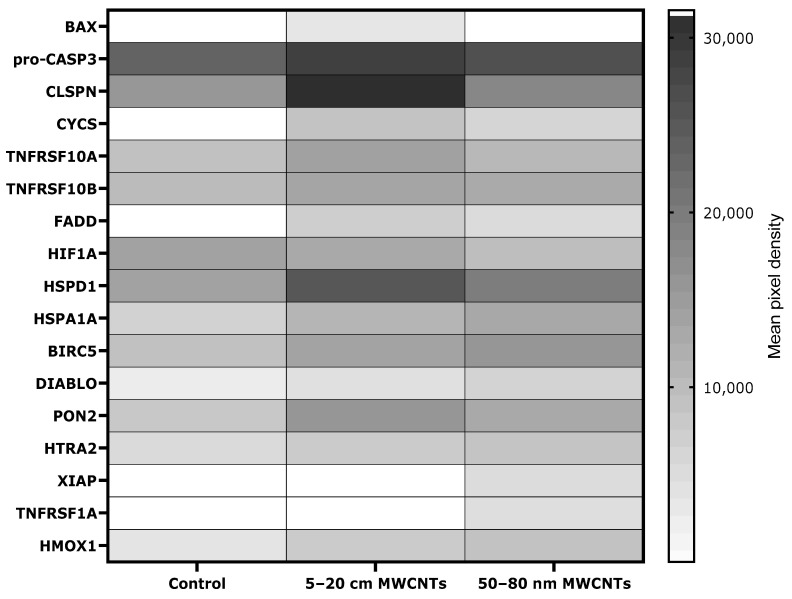
Expression of apoptosis-linked proteins measured in lysates of MDA-MB-436 cells treated with 50 µg/mL MWCNTs for 48 h. The semi-quantitative Proteome Profiler Human Apoptosis Array Kit was used in a mixture of cell lysates from three independent experiments. Protein levels are presented as a mean spot pixel density of two individual measurements, normalized to reference spots and subtracted for background control.

The Human Cytokine Array Kit (R&D Systems, Minneapolis, MN, USA) was used to assess the level of apoptosis-linked proteins. The array allowed for simultaneous determination of 35 proteins related to programmed cell death (Supplementary Materials Table S2). Using the profiler, we were able to demonstrate the presence of 17 apoptosis-linked proteins in MDA-MB-436 cells treated with MWCNTs. The analysis revealed that MWCNTs changed the secretion level of 12 of them: heme oxygenase 1 (HMOX1), paraoxonase 2 (PON2), diablo IAP-binding mitochondrial protein (DIABLO), baculoviral IAP repeat containing 5 (BIRC5), heat shock protein family D (Hsp60) member 1 (HSPD1), heat shock protein family A (Hsp70) member 1A (HSPA1A), hypoxia inducible factor 1 subunit alpha (HIF1A), TNF receptor superfamily member 10b (TNFRSF10B), TNF receptor superfamily member 10a (TNFRSF10A), cytochrome C (CYCS), claspin (CLSPN), and pro-caspase 3 (pro-CASP3). In addition, the presence of five proteins—BCL2-associated X, apoptosis regulator (BAX), cytochrome C (CYCS), X-linked inhibitor of apoptosis (XIAP), Fas-associated death domain (FADD), and TNF receptor superfamily member 1A (TNFRSF1A)—was induced by the treatment with MWCNTs, as these proteins were absent in the untreated control (Figure 8).

### 2.6. Impact of MWCNTs on Actin Filaments

In cells treated with MWCNTs, we observed primarily a reduction in the number of cells compared to untreated cells. In many living cells, serious disturbances of their actin cytoskeleton were visible. This was closely related to the previously demonstrated cytotoxicity. Nevertheless, in cells treated with the tested MWCNTs, a reduction in the number of cell clusters and an increase in the percentage of single cells was observed, but also, an elongated shape and the formation of actin clusters was observed at the ends of some of the cells (Supplementary Materials Figure S1).

## 3. Discussion

The relationship between exposure to NMs and the EMT process in cancers, although present in the literature, has not yet been adequately studied and requires further, extensive analysis. So far, such a relationship has been demonstrated for several types of metallic nanoparticles, e.g., titanium dioxide [9], copper oxide [14], nickel [15], cobalt [18], and silver [7,13,19]. Due to their profibrogenic properties, carbon-based nanomaterials should be analyzed in the context of EMT-mediated metastasis. Thus, the aim of our work was to assess the prometastatic potential of MWCNTs in relation to MDA-MB-436 breast cancer cells.

Existing studies proved the cytotoxicity of MWCNTs against cells derived from both normal and neoplastic lines. However, the assessment of cytotoxicity should be carried out individually for each experimental model due to significant deviations in results depending on, e.g., length and diameter, dose, surface area, level of dispersion in the cellular medium, and tendency to form agglomerates [20]. In our research, for a broader perspective, all experiments were carried out on two sizes of MWCNTs. It is generally assumed that biological activity of NMs increases as their size decreases, as smaller ones have a larger surface area on which interactions with cellular components can occur and the smaller size facilitates entry into cells. However, in the case of MWCNTs, which are a nanomaterial with two dimensions in the nanoscale, the results remain unclear. Some authors have shown that the cytotoxicity of MWCNTs decreases with the increase in diameter [21,22], while others have reported the opposite relationship [23]. Our studies have shown that both tested MWCNTs reduced the MDA-MB-436 cells viability in two assays differing in principle: the mitochondrial activity test (MTT) (Table 1) and the cytoplasmic membrane integrity test (NR) (Table 2). The present study showed the cytotoxic effect of non-functionalized MWCNTs on MDA-MB-436 cells for the first time in the literature; however, Fahrenholtz et. al. [24] demonstrated the cytotoxicity of MWCNTs functionalized with 1,2-distearoyl-sn-glycero-3-phosphoethanolamine with conjugated methoxy polyethylene glycol. Among TNBC lines with a low content of claudins, the most frequently used were MDA-MB-231-line cells, for which the cytotoxic effect of non-functionalized MWCNTs [25,26,27] and MWCNTs functionalized with polyethylene glycol [25], carboxyl groups [28], or chitosan [29] was demonstrated. In our studies, MDA-MB-436 cells were selected, which are capable of forming mammospheres during cultivation in non-adherent conditions and, compared to the MDA-MB-231 line, are less aggressive and characterized by reduced tumorigenic potential. 

The main mechanism of NM toxicity is their interaction with mitochondria, which leads to production of free radicals such as reactive oxygen (ROS) and/or nitrogen species. In line with this, ROS production is considered a major cause of MWCNT cytotoxicity [30]. However, some reports indicate that depending on the physicochemical parameters of MWCNTs, ROS generation may be insufficient for the explanation of MWCNTs’ overall cytotoxicity [23,31]. To the best of our knowledge, oxidative stress markers in MDA-MB-436 cells treated with MWCNTs have not yet been studied; however, results revealing direct induction of ROS by MWCNTs were obtained for MDA-MB-231 cells [26,28]. In our study, incubation of MDA-MB-436 cells with MWCNTs resulted in a distortion of the oxidative imbalance manifested by an increase in the MDA level (Figure 1) and a decrease in the free -SH groups (Figure 2).

As the main goal of our study was to assess the effect of MWCNTs on the metastatic potential of MDA-MB-436 cells, the expression of EMT markers, cell migration, and TGFB1 secretion were determined. We have shown that in MDA-MB-436 cells incubated with MWCNTs, the relative mRNA expression of mesenchymal markers *CDH2*, *VIM*, *MMP2*, and *MMP9* increased (Figure 3A–D), while the expression of epithelial *CDH1* decreased (Figure 3E). The obtained results indicated the EMT-stimulating effect of the tested MWCNTs. In line with this, MDA-MB-436 cell migration increased as a result of incubation with MWCNTs (Figure 4), but only to a small extent. Also, secretion of TGFB1, which is a key regulator of EMT and a strong profibrogenic factor, did not exceed 1.5 times the secretion noted for the control cells (Figure 5), which further confirms only a limited effect of the MWCNT treatment. In other TNBC models, while developing a MWCNT-coated scaffold for 3D culture of MDA-MB-231 cells, Akinoglu et al. showed an increase in the levels of mesenchymal EMT markers MMP9 and PIK3K kinase, which is a component of the EMT-related signaling pathway. However, the levels of AKT, MMP2, and NF-xK were similar to the control values [32]. Apparently, the impact of CNTs of EMT and the metastatic potential of cancer cell lines depends on nanotube characteristics and the cellular model used. In Graham et al.’s study, after culturing MDA-MB-231 cells on plates coated with collagen-functionalized MWCNTs, a slight increase in CDH1 expression and reduced migration at MWCNT concentrations of up to 20 µg/mL were shown, while the opposite trend was observed for higher MWCNT concentrations [33].

It is worth noting that the results obtained for other carbon-based NMs are ambiguous. MDA-MB-436 cells incubated with PEG-modified graphene oxide nanoplatelets showed reduced migration and invasion, likely due to impaired mitochondrial respiration and disruption of the actin cytoskeleton. However, the levels of EMT markers were not tested in this study [34]. Liu et al. showed that MDA-MB-231 cells incubated with gadoliniumfullurene were characterized by reduced migration and invasion, reduced expression of epithelial markers, and increased mesenchymal EMT markers. The authors suggest inhibition of TGF-β signaling as a mechanism. However, polyhydrolyzed fullurene alone used in the synthesis of gadoliniumfullurene had no significant effect on studied parameters [35]. Nevertheless, graphene and fullerenols are specific carbon NMs characterized by low toxicity and strong antioxidant properties, which most likely translates into the results obtained by the researchers [36]. In non-TNBC breast cancer models, MWCNTs did not change the level of migration of estrogen-dependent breast adenocarcinoma cells of the MCF7 line [37,38].

Interestingly, the EMT-stimulating effect of MWCNTs and other carbon NMs has been shown in several non-breast cancer cell models and normal cells. This effect was observed, e.g., in lung-derived cells of the non-cancerous BEAS-2B line [39,40,41] and cancerous A549 cells [6,11,42], in MeT-5A mesothelial cells [43,44], and in prostate PC3 cancer cells [45]. Furthermore, in vivo studies have shown that CNTs are capable of inducing pulmonary [5,6,46,47,48,49], pleural [50], and liver [51,52] fibrosis, which is related directly to the EMT subtype II mechanism. Lu et al. showed that MWCNTs promote breast cancer metastasis to the lung in a mouse model by inducing a generalized inflammatory process accompanied by an increase in the levels of prometastatic and proangiogenic factors [53]. As a mechanism of MWCNTs induced metastasis promotion, activation of the TGF-β1/Smad2 signaling pathway [6,40] or Smad-independent TGF-β1/AKT/GSK-3β pathway [39] was proposed. In our studies, however, as already mentioned, TGFB1 secretion as a result of MWCNT treatment increased only modestly compared to control cells. It is therefore possible that other mechanisms may be responsible for the increased expression of mesenchymal EMT markers in the studied system. Although the TGF-β1 signaling pathway is crucial for EMT, many others have been described to be involved in this process, e.g., Wnt, Notch, Hedgehog, RTK, and TNF-alpha [54]. The obtained results are ambiguous and do not sufficiently explain the mechanism that causes the increase in the level of a number of mesenchymal markers as a result of treatment of MDA-MB-436 cells with MWCNTs. In the future, it would be worthwhile to focus on a broader analysis of EMT signaling pathway proteins, assess the level of β-catenin phosphorylation and its possible intracellular translocation, and also examine the expression of EMT transcription factors.

The tumor microenvironment is closely related to metastasis. Therefore, the impact of MWCNTs on the secretion of pro-inflammatory proteins by MDA-MB-436 cells was determined. Our study has shown that MDA-MB-436 cells treated with MWCNTs secreted an increased amount of several proteins that are essential for breast cancer metastasis (Figure 6). In cells treated with MWCNTs, increased secretion of IL6 and CXCL8, the two cytokines of essential importance in EMT, was observed, which stimulate it via a feedback loop interaction. CXCL8 binds to CXCR1 and CXCR2 receptors and activates several EMT-related signaling pathways, including PI3K-Akt and MAPK [55]. However, the potential of IL6 to induce and maintain EMT in breast cancer cell lines depends on activation of the JAK/STAT3 pathway [56]. Furthermore, the profile of pro-inflammatory proteins secreted by MDA-MB-436 cells treated with MWCNTs (primarily IL6, CXCL8, and CCL5) mimics the action of tumor-associated macrophages (TAMs), the largest population of inflammatory cells in the tumor stroma, associated with tumor progression, EMT stimulation, and promotion of metastasis [57]. At the same time, CXCL8, CXCL1, CCL5, and MIF may act as recruitment factors that enable the recruitment of monocytes/macrophages and their polarization to TAMs [58]. Moreover, it has been shown that MDA-MB-436 cells treated with MWCNTs produce a chemotactic gradient of CXCL1 and CXCL8, which may act as a potential attractant for myeloid-derived suppressor cells. These cells may in turn activate signaling pathways associated with EMT [59]. In line with this, MWCNTs have been shown to induce a pro-inflammatory response in immune cells, such as macrophages, monocytes, and dendritic cells in vitro [60,61,62]. Sustained inflammation has also been demonstrated in animal models as a result of CNT treatment [63]. Our results indicate that MWCNTs increase the secretion of a number of cytokines and chemokines of key importance in the metastasis process.

The next stage of research was to determine the level of apoptosis markers, since it has been shown that apoptosis is suppressed in cancer cells during EMT, which facilitates the induction of cancer stem cells, enhances immunosuppression in the tumor microenvironment, and stimulates the angiogenesis process [64]. Our results revealed that the expression of a number of proapoptotic proteins increased in MDA-MB-436 cells treated with MWCNTs, which indicates that some cells entered the programmed death pathway as a result of exposure (Figure 8). The observed increase in pro-apoptotic proteins was expected and corresponds to observed MWCNT cytotoxicity. This is in line with other studies conducted on MDA-MB-231 cells [28,29]. Interestingly, in MWCNT-treated MDA-MB-436 cells, we observed an increase in the expression of several potentially anti-apoptotic proteins (HSPD1, HSPA1A, PON2, HMOX1, XIAP, BIRC5, and CLSPN). Thus, it seems that the faith of cells exposed to MWCNTs is a matter of a balance between anti- and proapoptotic signals, likely depending on the actual state of the cell. The appearance of antiapoptotic mechanisms has been demonstrated for different NMs and various cellular models, e.g., silver, zinc, or selenium nanoparticles [65,66,67]. Furthermore, development of anti-apoptotic mechanisms by cancer cells that have entered the EMT process may be an effect of avoidance of anoikis [68,69,70] or entering anastasis [71,72].

Finally, we would like to emphasize once again that the results of studies on the biological activity of CNTs should be assessed individually. Sometimes it is very problematic to compare the results of individual experiments. The source of discrepancies between the results of individual studies are primarily the physicochemical parameters of the tested materials. CNTs are an extremely heterogeneous group, which differ, for example, in length, shape, or type of metal impurity, which makes risk assessment difficult. A very important feature of MWCNTs, determining their nature and influence on biological structures, is also the type of surface functionalization. Moreover, the final cellular response may change as a result of aggregation and agglomeration of the tested materials. Moreover, MWCNTs may exhibit structural defects, which may affect the increased induction of oxidative stress and cytotoxicity [73,74,75].

In summary, we have studied for the first time the effect of MWCNTs on TNBC cells of the MDA-MB-436 line. We have shown that MWCNTs reduce cell viability and induce oxidative stress, inflammation, and apoptosis. These optimistic results, which could potentially indicate an anti-cancer effect of the studied MWCNTs, are in contrast to further results indicating a potentially prometastatic effect. A significant increase in the level of mesenchymal markers of the EMT process was observed, although the level of migration increased only slightly. An increase in the level of several antiapoptotic proteins was also noted, and some of the pro-inflammatory proteins secreted by the studied cells have a proven role in the metastasis process. The obtained results, although ambiguous, indicate that CNTs have the potential to stimulate metastasis via the EMT process. The effect of MWCNTs on MDA-MB-436 breast cancer cells is multifaceted and requires further, detailed analysis, especially due to the dynamic development of nanotechnology and the fact that cancer is the second leading cause of death in the world.

## 4. Materials and Methods

### 4.1. MDA-MB-436 Cell Culture

All experiments were performed on MDA-MB-436 cells purchased from Cell Line Service (CLS, Hamburg, Germany). These triple-negative breast cancer cells derive from pleural effusions of infiltrating ductal carcinoma. The cells belong to the molecular subtype with low claudin expression. The cells were grown in a 1:1 mixture of DMEM and Ham’s F-12 media supplemented with L-glutamine, penicillin–streptomycin, and 10% fetal bovine serum (all reagents were purchased from Sigma Aldrich, Saint Louis, MO, USA).

### 4.2. MWCNT Preparation

Commercially available, pristine, not functionalized MWCNTs with two outer diameters—5–20 nm and 50–80 nm—were bought from PlasmaChem GmbH (Berlin, Germany). For a more detailed description, see Supplementary Materials Table S3. MWCNT stock solutions (2 mg/cm^3^) were prepared by the dispersion of 2 mg of MWCNTs in 800 µL of distilled water. Then, 100 µL of phosphate-buffered saline (10×) and 100 µL of 15% bovine serum albumin solution were added. In order to achieve an optimal MWCNT dispersion in the cell culture media prior to addition to cell cultures, all solutions were sonicated on ice using a VCX 130 ultrasonic processor (Sonics & Materials Inc., Newton, CT, USA). After sonication, intermediate dilutions were immediately carried out by diluting the stock solution in an appropriate cell medium.

### 4.3. MTT Assay

MDA-MB-436 cells were plated on 96-well microplates (TPP, Trasadingen, Switzerland) at a density of 1 × 10^4^ cells/well in 100 µL of culture medium. The cells were allowed to grow for 24 h, and culture medium was replaced with the same medium containing MWCNTs at a final concentration in the range of 0–100 µg/mL for 24 h or 48 h. Then, 15 µL of 3-(4,5-dimethylthiazol-2-yl)-2,5-diphenyltetrazolium bromide (MTT) of 5 mg/mL stock solution were added to each well and incubated for 3 h at 37 °C. After incubation, the fluid was poured out and 100 µL of dimethyl sulfoxide were added to each well and vigorously shaken for 2 min. Finally, absorbance was read at 570 nm using a FLUOstar Omega multi-mode microplate reader (BMG LABTECH, Ortenberg, Germany).

### 4.4. Neutral Red Assay

MDA-MB-436 cells were plated on 96-well microplates (TPP, Trasadingen, Switzerland) at a density of 2 × 10^4^ cells/well in 200 µL of culture medium. The cells were allowed to grow overnight, and the culture medium was replaced with the same medium containing MWCNTs at a final concentration in the range of 0–100 µg/mL for 24 h or 48 h. After treatment, the MWCNT solutions were aspirated, and the cells were washed with 150 μL of PBS and incubated with 100 μL of 50 μg/mL neutral red (NR) solution for 4 h at 37 °C. After removing the NR solution, the cells were washed with PBS (150 μL), and 200 μL of 50% ethanol: 49% water: 1% acetic acid solution were added to each well. Absorbance was red at 540 nm after 20 min of rocking using an FLUOstar Omega multi-mode microplate reader (BMG LABTECH, Ortenberg, Germany).

### 4.5. Sample Preparation for Analysis

Cells of the MDA-MB-436 line (2 × 10^5^ cells/mL) were seeded into 6-well plates and incubated overnight to reached an appropriate level of attachment to the surface. The next day, the cells were treated with freshly prepared appropriate MWCNT solutions in the culture medium. Medium without the addition of MWCNTs was used as a control. After 24 or 48 h of treatment, the supernatant was collected and frozen for further experiments. The cells were then washed with PBS, trypsinized, harvested, and centrifugated (12,000× *g*) for 10 min at 4 °C. Cell pellet was washed twice with PBS and frozen at −80 °C for subsequent analysis. Cell lysates were made by suspending the cell pellets in RIPA buffer additionally containing a cocktail of protease and phosphatase inhibitors (both Thermo Fisher Scientific Inc., Waltham, MA, USA).

### 4.6. QuantiChromTM TBARS Assay Kit

The level of malondialdehyde in MDA-MB-436 cells lysates was determined using a commercially available QuantiChrom^TM^ TBARS Assay Kit (BioAssay Systems, Hayward, CA, USA). The test was performed according to the instructions provided by the manufacturer, and the amount of reaction product was determined spectrophotometrically (OD 535 nm) using a FLUOstar Omega multi-mode microplate reader (BMG LABTECH, Ortenberg, Germany).

### 4.7. Fluorometric Thiol Assay Kit

The level of free thiol groups in MDA-MB-436 cells lysates was determined using a commercially available Fluorometric Thiol Assay Kit (Sigma-Aldrich, Saint Louis, MO, USA), according to the instructions provided by the manufacturer. A FLUOstar Omega multi-mode microplate reader (BMG LABTECH, Ortenberg, Germany) was used for fluorimetric analysis of the obtained reaction product (λex = 490/λem = 535 nm).

### 4.8. Proteome Profiler Human Cytokine Array Kit and Proteome Profiler Human Apoptosis Array Kit

The conditioned media from three independent experiments were pooled together and spotted on membranes of the Proteome Profiler Human Cytokine Array Kit, while similarly mixed cell lysates were used to employ the Proteome Profiler Human Apoptosis Array Kit (both from R&D Systems, Minneapolis, MN, USA). The membranes were developed as recommended by the manufacturer. The resulting spots were visualized using a Carestream^®^ BioMax^®^ (Kodak, Rochester, NY, USA). Image J 1.54 software (National Institutes of Health, Bethesda, MD, USA) was used to quantify the intensity of specific spots. The average of the two spots corresponding to the same protein was calculated and the value corresponding to the intensity of background was subtracted. Protein intensity was then normalized to reference points, included in the test by a producent to align the transparency overlay template and to demonstrate that the array had been incubated with Streptavidin-HRP during the assay procedure. Both profilers were used to assess the expression of proteins in the experiments where the cells were incubated for 48 h with 50 µg/mL MWCNTs.

### 4.9. ELISA

The Human IL-6 ELISA Kit, Human TGF beta 1 Kit (both Sigma-Aldrich, Saint Louis, MO, USA), and Human IL-8 ELISA Kit (Abcam, Cambridge, UK) were used to determine the level of IL6, TGFB1, and CXCL8 secretion in the culture media of MDA-MB-436 cells treated with MWCNTs. The kits were used as recommended by the manufacturers.

### 4.10. Real-Time Reverse Transcription Polymerase Chain Reaction

*CDH2*, *VIM*, *CDH1*, *MMP2*, and *MMP9* mRNA expression analysis in MWCNT-treated MDA-MB-436 cells after 24 h of incubation was performed using qRT-PCR. Total RNA was isolated using a high-purity RNA isolation kit (Roche, Basel, Switzerland), as recommended by the manufacturer. The quality and quantity of isolated RNA was measured using a NanoDrop^®^ spectrophotometer (Thermo Fisher Scientific, Inc.). Reverse transcription was performed using a high-capacity cDNA reverse transcriptase (Thermo Fisher Scientific, Inc.). qRT-PCR was performed using an Applied Biosystems^®^ 7500 Fast Real-Time PCR System thermal cycler (Applied Biosystems, Waltham, MA, USA). For optimal results, PowerUp SYBR™ Green Master Mix (Thermo Fisher Scientific, Inc.) and Dual-Lock Taq DNA polymerase were used. Primer pairs specific for human *CDH2*, *VIM*, *MMP2*, *MMP9*, *CDH1*, and β-actin (*ACTB*) were purchased from Sino Biological Inc. (Beijing, China), Cat No. HP100018, HP100124, HP100168, HP100367, and HP100067, respectively. Relative expression of mRNA was calculated using the 2^−ΔΔCt^ method with *ACTB* as an endogenous control to normalize the expression of the studied markers.

### 4.11. Chemotaxis Cell Migration Assay

To determine the effect of the tested MWCNTs on the relative level of migration of MDA-MB-436 cells after 24 h of incubation, the QCM^TM^ Chemotaxis Cell Migration Assay (Sigma-Aldrich, Saint Louis, MO, USA) was used as recommended by the manufacturer. This assay is performed in a migration chamber that is based on the Boyden chamber principle. The 5 μm pores in the chamber membrane are suitable for testing the migration rate of cancer cells such as MDA-MB-436.

### 4.12. Phalloidin–TRITC Staining

The MDA-MB-436 cells were cultured on 8-well Nunc^®^ Lab-Tek^®^ Chamber Slide™ culture slides (Thermo Fisher Scientific Inc., Waltham, MA, USA) for 24 h and then treated with appropriate solutions of the tested MWCNTs. After 48 h, the cells were fixed with 3.7% paraformaldehyde and permeabilized with 0.1% Triton X. Phalloidin–tetramethylrhodamine B isothiocyanate (Sigma Aldrich, Saint Louis, MO, USA) was used to stain actin filaments, and DAPI was used to stain cell nuclei (both Sigma Aldrich, Saint Louis, MO, USA). Images were taken with an Olympus BX51 fluorescence microscope (Olympus Corporation, Tokyo, Japan).

### 4.13. Statistical Analysis

Statistical analysis of the obtained results was performed using GraphPad Prism 9.0 software (GraphPad Software Inc., San Diego, CA, USA). For all analyses, at least three independent experiments were included. The obtained results were expressed as mean ± standard deviation. The Shapiro–Wilk test confirmed the normality of the distribution of the analyzed data. Then, statistical significance was assessed using one-way ANOVA. Tukey’s test was applied for post hoc comparison. Differences were defined as statistically significant when *p* < 0.05. Semi-quantitative results of the Proteome Profiler Human Cytokine Array and Proteome Profiler Human Apoptosis Array assays were excluded from the statistical analysis. All graphs presented in the manuscript were prepared using GraphPad Prism 9.0 software.

## Figures and Tables

**Table 1:** Effect of MWCNTs on MDA-MB-436 cell viability measured by MTT assay.

MTT				
	5–20 nm MWCNTs		50–80 nm MWCNTs	
	24 h	48 h	24 h	48 h
10 µg/mL	81 ± 6 *	73 ± 4 *	90 ± 3	70 ± 4 *
25 µg/mL	67 ± 4 *	67 ± 5 *	78 ± 6 *	60 ± 5 *
50 µg/mL	67 ± 6 *	60 ± 6 *	69 ± 7 *	56 ± 6 *
100 µg/mL	69 ± 5 *	57 ± 5 *	63 ± 8 *	48 ± 3 *

* Data are presented as mean of percentage of untreated control ± SD (*n* = 3). The asterisk denotes statistical significance at *p* < 0.05.

**Table 2:** Effect of MWCNTs on MDA-MB-436 cell viability measured by NR assay.

NR				
	5–20 nm MWCNTs		50–80 nm MWCNTs	
	24 h	48 h	24 h	48 h
10 µg/mL	83 ± 1 *	83 ± 3 *	96 ± 2	94 ± 3
25 µg/mL	80 ± 4 *	80 ± 5 *	88 ± 7	85 ± 4
50 µg/mL	80 ± 6 *	81 ± 1 *	88 ± 4	74 ± 4 *
100 µg/mL	72 ± 5 *	67 ± 4 *	83 ± 7 *	67 ± 11 *

* Data are presented as mean of percentage of untreated control ± SD (*n* = 3). The asterisk denotes statistical significance at *p* < 0.05.

## Data Availability

All data are contained within the article/Supplementary Materials. Raw data points from the study are available on request from the corresponding author.

## Supplementary Materials

The following supporting information can be downloaded at: https://www.mdpi.com/article/10.3390/ijms26062777/s1.

## References

[1] Rathinavel S., Priyadharshini K., Panda D. (2021). A review on carbon nanotube: An overview of synthesis, properties, functionalization, characterization, and the application. Mater. Sci. Eng. B.

[2] Francis A.P., Devasena T. (2018). Toxicity of carbon nanotubes: A review. Toxicol. Ind. Health..

[3] Awasthi S., Srivastava A., Kumar D., Pandey S.K., Mubarak N.M., Dehghani M.H., Ansari K. (2024). An insight into the toxicological impacts of carbon nanotubes (CNTs) on human health: A review. Environ. Adv..

[4] Yuan X., Zhang X., Sun L., Wei Y., Wei X. (2019). Cellular Toxicity and Immunological Effects of Carbon-based Nanomaterials. Part. Fibre Toxicol..

[5] Chang C.C., Tsai M.L., Huang H.C., Chen C.Y., Dai S.X. (2012). Epithelial-mesenchymal transition contributes to SWCNT-induced pulmonary fibrosis. Nanotoxicology.

[6] Chen T., Nie H., Gao X., Yang J., Pu J., Chen Z., Cui X., Wang Y., Wang H., Jia G. (2014). Epithelial–mesenchymal transition involved in pulmonary fibrosis induced by multi-walled carbon nanotubes via TGF-beta/Smad signaling pathway. Toxicol. Lett..

[7] Rakowski M., Porębski S., Grzelak A. (2021). Silver Nanoparticles Modulate the Epithelial-to-Mesenchymal Transition in Estrogen-Dependent Breast Cancer Cells In Vitro. Int. J. Mol. Sci..

[8] Dhawan U., Sue M.W., Lam K.C., Buddhakosai W., Huang P.H., Chen Y.C., Chen P.C., Chen W.L. (2018). Nanochip-Induced Epithelial-to-Mesenchymal Transition: Impact of Physical Microenvironment on Cancer Metastasis. ACS Appl. Mater. Interfaces.

[9] Setyawati M.I., Sevencan C., Bay B.H., Xie J., Zhang Y., Demokritou P., Leong D.T. (2018). Nano-TiO2 Drives Epithelial–Mesenchymal Transition in Intestinal Epithelial Cancer Cells. Small.

[10] Sahu S.C., Zheng J., Yourick J.J., Sprando R.L., Gao X. (2015). Toxicogenomic responses of human liver HepG2 cells to silver nanoparticles. J. Appl. Toxicol..

[11] Ventura X., Pereira J.F.S., Matos P., Marques B., Jordan P., Sousa-Uva A., Silva M.J. (2020). Cytotoxicity and genotoxicity of MWCNT-7 and crocidolite: Assessment in alveolar epithelial cells versus their coculture with monocyte-derived macrophages. Nanotoxicology.

[12] Wang D.P., Shen J., Qin C.Y., Li Y.M., Gao L.J., Zheng J., Feng Y.L., Yan Z., Zhou X., Cao J.M. (2022). Platinum nanoparticles promote breast cancer cell metastasis by disrupting endothelial barrier and inducing intravasation and extravasation. Nano Res..

[13] Matysiak-Kucharek M., Sawicki K., Kurzepa J., Wojtyła-Buciora P., Kapka-Skrzypczak L. (2023). The influence of silver nanoparticles on the proces of epithelial-mesenchymal transition in the context of cancer metastases. Med. Pr. Work. Health Saf..

[14] Zhang Y., Mo Y., Yan J., Zhang Y., Mo L., Zhang Q. (2021). MMP-3 activation is involved in copper oxide nanoparticle-induced epithelial-mesenchymal transition in human lung epithelial cells. Nanotoxicology.

[15] Yuan J., Mo Y., Zhang Y., Zhang Y., Zhang Q. (2022). Nickel nanoparticles induce epithelial-mesenchymal transition in human bronchial epithelial cells via the HIF-1α/HDAC3 pathway. Nanotoxicology.

[16] Lüönd F., Sugiyama N., Bill R., Bornes L., Hager C., Tang F., Santacroce N., Beisel C., Ivanek R., Burglin T. (2021). Distinct contributions of partial and full EMT to breast cancer malignancy. Dev. Cell.

[17] Hashemi M., Arani H.Z., Orouei S., Fallah S., Ghorbani A., Khaledabadi M., Kakavand A., Tavakolpournegari A., Saebfar H., Heidari H. (2022). EMT mechanism in breast cancer metastasis and drug resistance: Revisiting molecular interactions and biological functions. Biomed. Pharmacother..

[18] Yuan J., Mo Y., Zhang Y., Zhang Y., Zhang Q. (2024). HMGB1 derived from lung epithelial cells after cobalt nanoparticle exposure promotes the activation of lung fibroblasts. Nanotoxicology.

[19] Matysiak-Kucharek M., Czajka M., Jodłowska-Jędrych B., Sawicki K., Wojtyła-Buciora P., Kruszewski M., Kapka-Skrzypczak L. (2020). Two Sides to the Same Coin—Cytotoxicity vs. Potential Metastatic Activity of AgNPs Relative to Triple-Negative Human Breast Cancer MDA-MB-436 Cells. Molecules.

[20] Zhou L., Forman H.J., Ge Y., Lunec J. (2017). Multi-walled carbon nanotubes: A cytotoxicity study in relations to functionalization, dose and dispertion. Toxicol. Vitr..

[21] Zhao X., Chang S., Long J., Li J., Li X., Cao Y. (2019). The toxicity of multi-walled carbon nanotubes (MWCNTs) to human endothelial cells: The influence of diameters of MWCNTs. Food Chem. Toxicol..

[22] Fröhlich E., Meindl C., Höfler A., Leitinger G., Roblegg E. (2012). Combination of small size and carboxyl functionalisation causes cytotoxicity of short carbon nanotubes. Nanotoxicology.

[23] Fuijta K., Obara S., Maru J., Endoh S. (2020). Cytotoxicity profiles of multi-walled carbon nanotubes with diffrent physico-chemical properties. Toxicol. Mech. Methods.

[24] Fahrenholtz C.D., Ding S., Bernish B.W., Wright M.L., Zheng Y., Yang M., Yao X., Donati G.L., Gross M.D., Bierbach U. (2016). Design and cellular studies of a carbon nanotube-based delivery system for a hybrid platinum-acridine anticancer agent. J. Inorg. Biochem..

[25] Sharma S., Naskar S., Kuotsu K. (2020). Metronomic chemotherapy of carboplatin-loaded PEGylated MWCNTs: Synthesis, characterization and in vitro toxicity in human breast cancer. Carbon Lett..

[26] Badea N., Craciun M.M., Drogomir A.S., Balas M., Dinischiotu A., Nistor C., Gavan C., Ionita D. (2020). Systems based on carbon nanotubes with potential in cancer therapy. Mater. Chem. Phys..

[27] Ozgen P.S.O., Atasoy S., Kurt B.Z., Durmus Z., Yigit G., Dag A. (2020). Glycopolymer decorated multiwalled carbon nanotubes for dual targeted breast cancer therapy. J. Mater. Chem. B.

[28] Badea M.A., Prodana M., Dinischiotu A., Crihana C., Ionita D., Balas M. (2018). Cisplatin Loaded Multiwalled Carbon Nanotubes Induce Resistance in Triple Negative Breast Cancer Cells. Pharmaceutics.

[29] Kavousi M., Chavoshi M.S. (2020). Effect of coated carbon nanotubes with chitosan and cover of flaxseed in the induction of MDA-MB-231 apoptosis by analyzing the expression of Bax and Bcl-2. Meta Gene.

[30] Salih S.J., Ghobadi M.Z. (2022). Evaluating the cytotoxicity and pathogenicity of multi-walled carbon nanotube through weighted gene co-expression network analysis: A nanotoxicogenomics study. BMC Genom. Data.

[31] Sabido O., Figarol A., Klein J.P., Bin V., Forest V., Pourchez J., Fubini B., Cottier M., Tomatis M., Boudard D. (2020). Quantitative Flow Cytometric Evaluation of Oxidative Stress and Mitochondrial Impairment in RAW 264.7 Macrophages after Exposure to Pristine, Acid Functionalized, or Annealed Carbon Nanotubes. Nanomaterials.

[32] Akinoglu E.M., Ozbilgin K., Sonmez P.K., Ozkut M.M., Giersig M., Inan S., Gumustepe E., Kurtman C. (2017). Biocompatibility of vertically aligned multi-walled carbon nanotube scaffolds for human breast cancer cell line MDA-MB-231. Prog. Biomater..

[33] Graham E.G., Wailes E.M., Levi-Polyachenko N.H. (2016). Multi-Walled Carbon Nanotubes Inhibit Breast Cancer Cell Migration. J. Biomed. Nanotechnol..

[34] Zhou H., Zhang B., Zheng J., Yu M., Zhou T., Zhao K., Jia Y., Gao X., Chen C., Wei T. (2014). The inhibition of migration and invasion of cancer cells by graphene via the impairment of mitochondrial respiration. Biomaterials.

[35] Liu Y., Chen C., Qian P., Lu X., Sun B., Zhang X., Wang L., Gao X., Li H., Chen Z. (2015). Gd-metallofullerenol nanomaterial as non-toxic breast cancer stem cell-specific inhibitor. Nat. Commun..

[36] Gaur M., Misra C., Yadav A.B., Swaroop S., Maolmhuaidh F., Bechelany M., Barhoum A. (2021). Biomedical Applications of Carbon Nanomaterials: Fullerenes, Quantum Dots, Nanotubes, Nanofibers, and Graphene. Materials.

[37] Garcia-Hevia L., Valiente R., Fernandez-Luna J.L., Flahaut E., Rodriquez-Fernandez L., Villegas J.C., Gonzales J., Fanarraga M.L. (2015). Inhibition of Cancer Cell Migration by Multiwalled Carbon Nanotubes. Adv. Health Mater..

[38] Yao H.J., Zhang Y.G., Sun L., Liu Y. (2014). The effect of hyaluronic acid functionalized carbon nanotubes loaded with salinomycin on gastrin cancer stem cells. Biomaterials.

[39] Polimeni M., Gulino G.R., Gazzano E., Kopecka J., Marucco A., Fenoglio I., Cesano F., Campagnolo L., Magrini A., Pietroiusti A. (2016). Multi-walled carbon nanotubes directly induce epithelial-mesenchymal transition in human bronchial epithelial cells via the TGF-β-mediated Akt/GSK-3β/SNAIL-1 signalling pathway. Part. Fibre Toxicol..

[40] Chen P., Tian K., Tu W., Zhang Q., Han L., Zhou X. (2019). Sirtuin 6 inhibits MWCNTs-induced epithelial-mesenchymal transition in human bronchial epithelial cells via inactivating TGF-β1/Smad2 signaling pathway. Toxicol. Appl. Pharmacol..

[41] Wang P., Voronkova M., Luanpitpong S., He X., Riedel H., Dinu C.Z., Wang L., Rojanasakul Y. (2017). Induction of Slug by Chronic Exposure to Single-Walled Carbon Nanotubes Promotes Tumor Formation and Metastasis. Chem. Res. Toxicol..

[42] Ju L., Zhang G., Zhang X., Jia Z., Gao X., Jiang Y., Yan C., Duerksen-Hughes P.J., Chen F.F., Li H. (2014). Proteomic Analysis of Cellular Response Induced by Multi-Walled Carbon Nanotubes Exposure in A549 Cells. PLoS ONE.

[43] Lohcharoenkal W., Wang L., Stueckle T.A., Park J., Tse W., Dinu C.Z., Rojanasakul Y. (2014). Role of H-Ras/ERK signaling in carbon nanotube-induced neoplastic-like transformation of human mesothelial cells. Front. Physiol..

[44] Turini S., Bergandi L., Gazzano E., Prado M., Alderi E. (2019). Epithelial to Mesenchymal Transition in Human Mesothelial Cells Exposed to Asbestos Fibers: Role of TGF-β as Mediator of Malignant Mesothelioma Development or Metastasis via EMT Event. Int. J. Mol. Sci..

[45] Zhu J., Li B., Xu M., Liu R., Xia T., Zhang Z., Xu Y., Liu S. (2020). Graphene Oxide Promotes Cancer Metastasis through Associating with Plasma Membrane to Promote TGF-β Signaling-Dependent Epithelial–Mesenchymal Transition. ACS Nano.

[46] Vetti G., Lison D., Van den Brule S. (2016). Mechanisms of lung fibrosis induced by carbon nanotubes: Towards an Adverse Outcome Pathway (AOP). Part. Fibre Toxicol..

[47] Dong J., Ma Q. (2016). Myofibroblasts and lung fibrosis induced by carbon nanotube exposure. Part. Fibre Toxicol..

[48] Dong J., Ma Q. (2018). Type 2 Immune Mechanisms in Carbon Nanotube-Induced Lung Fibrosis. Front. Immunol..

[49] Duke K.S., Bonner J.C. (2018). Mechanisms of carbon nanotube-induced pulmonary fibrosis: A physicochemical characteristic perspective. WIREs Nanomed. Nanobiotechnol..

[50] Arnoldussen Y.J., Skaug V., Aleksandersen M., Ropstad E., Anmarkrud K.H., Einarsdottir E., Chin-Lin F., Bjorklund C.G., Kasem M., Eilertsen E. (2018). Inflammation in the pleural cavity following injection of multi-walled carbon nanotubes is dependent on their characteristics and the presence of IL-1 genes. Nanotoxicology.

[51] Kim J.E., Lee S., Lee A.Y., Seo H.W., Chae C., Cho M.H. (2015). Intratracheal exposure to multi-walled carbon nanotubes induces a nonalcoholic steatohepatitis-like phenotype in C57BL/6J mice. Nanotoxicology.

[52] Liu E., Wang X., Li X., Tian P., Xu H., Li Z., Wang L. (2020). Co-exposure to multi-walled carbon nanotube and lead ions aggravates hepatotoxicity of nonalcoholic fatty liver via inhibiting AMPK/PPARγ pathway. Aging.

[53] Lu X., Zhu Y., Bai R., Wu Z., Qian W., Yang L., Cai R., Yan H., Li T., Pandey V. (2019). Long-term pulmonary exposure to multi-walled carbon nanotubes promotes breast cancer metastatic cascades. Nat. Nanotechnol..

[54] Liaghat M., Ferdousmakan S., Mortazavi S.H., Yahyazadeh S., Irani A., Banihashemi S., Asl F.S.S., Akbari A., Farzam F., Aziziyan F. (2024). The impact of epithelial-mesenchymal transition (EMT) induced by metabolic processes and intracellular signaling pathways on chemo-resistance, metastasis, and recurrence in solid tumors. Cell Commun. Signal..

[55] Deng F., Weng Y., Li X., Wang T., Fan M., Shi Q. (2021). Overexpression of IL-8 promotes cell migration via PI3K-Akt signaling pathway and EMT in triple-negative breast cancer. Pathol. Res. Pract..

[56] Manore S.G., Doheny D.L., Wong G.L., Lo H.W. (2022). IL-6/JAK/STAT3 Signaling in Breast Cancer Metastasis: Biology and Treatment. Front. Oncol..

[57] Keeley T., Costanzo-Garvey D.L., Cook L.M. (2019). Unmasking the Many Faces of Tumor-Associated Neutrophils and Macrophages: Considerations for Targeting Innate Immune Cells in Cancer. Trends Cancer.

[58] Yang Q., Guo N., Zhou Y., Chen J., Wei Q., Han M. (2020). The role of tumor-associated macrophages (TAMs) in tumor progression and relevant advance in targeted therapy. Acta Pharm. Sin. B.

[59] Cai J., Cui Y., Yang J., Wang S. (2021). Epithelial-mesenchymal transition: When tumor cells meet myeloid-derived supressor cells. Biochim. Biophys. Acta (BBA) Rev. Cancer.

[60] Li Y., Cao J. (2018). The impact of multi-walled carbon nanotubes (MWCNTs) on macrophages: Contribution of MWCNT characteristics. Sci. China Life Sci..

[61] Ma J., Li R., Qu G., Liu H., Yan B., Xia T., Liu Y., Liu S. (2016). Carbon nanotubes stimulate synovial inflammation by inducing systemic pro-inflammatory cytokines. Nanoscale.

[62] Dong X., Liu L., Zhu D., Zhang H., Li Y., Leng X. (2015). Effects of Carboxylated Multiwalled Carbon Nanotubes on the Function of Macrophages. J. Nanomater..

[63] Kobayashi N., Izumi H., Morimoto Y. (2017). Review of toxicity studies of carbon nanotubes. J. Occup. Health.

[64] Wang G., Xu D., Zhang Z., Li X., Shi J., Sun J., Liu H.Z., Li X., Zhou M., Zheng T. (2021). The pan-cancer landscape of crosstalk between epithelial-mesenchymal transition and immune evasion relevant to prognosis and immunotherapy response. npj Precis. Oncol..

[65] Brzóska K., Męczyńska-Wielgosz S., Stępkowski T.M., Kruszewski M. (2015). Adaptation of HepG2 cells to silver nanoparticles-induced stress is based on the pro-proliferative and anti-apoptotic changes in gene expression. Mutagenesis.

[66] Hosseini A., Baeeri M., Rahimifard M., Navaei-Nigjeh M., Mohammadirad A., Pourkhalili N., Hassani S., Kamali M., Abdollahi M. (2013). Antiapoptotic effects of cerium oxide and yttrium oxide nanoparticles in isolated rat pancreatic islets. Hum. Exp. Toxicol..

[67] Goncalves D.M., Girard D., Goncalves D. (2014). Zinc oxide nanoparticles delay human neutrophilapoptosis by a de novo protein synthesis-dependent and reactive oxygen species-independent mechanism. Toxicol. Vitr..

[68] Cao Z., Livas T., Kyprianou N. (2016). Anoiks and EMT: Lethal “Liaisons” during Cancer Progression. Crit. Rev. Oncog..

[69] Assani G., Zhou Y. (2018). Effect of modulation of epithelial mesenchymal transition regulators Snail1 and Snail2 on cancer cell radiosensitivity by targeting of the cell cycle, cell apoptosis and cell migration/invasion. Oncol. Lett..

[70] Paoli P., Giannoni E., Chiarugi P. (2013). Anoikis molecular pathways and its role in cancer progression. Biochim. Biophys. Acta (BBA) Mol. Cell Res..

[71] Chakraborty S., Mir K.B., Seligson N.D., Nayak D., Kumar R., Goswami A. (2020). Integration of EMT and cellular survival instincts in reprogramming of programmed cell death to anastasis. Cancer Metastasis Rev..

[72] Sun G., Guzman E., Balasanyan V., Conner C.M., Wong K., Zhou H.R., Kosik K.S., Montell D.J. (2017). A molecular signature for anastasis, recovery from the brink of apoptotic cell death. J. Cell Biol..

[73] Johnston H.J., Hutchison G.R., Christensen F.M., Peters S., Hankin S., Aschberger K., Stone V. (2010). A critical review of the biological mechanisms underlying the in vivo and in vitro toxicity of carbon nanotubes: The contribution of physico-chemical characteristics. Nanotoxicology.

[74] Bengalli R.D., Zerbi G., Lucotti A., Catelani T., Mantecca P. (2023). Carbon nanotubes: Structural defects as stressors inducing lung cell toxicity. Chem. Biol. Interact..

[75] Muller J., Huaux F., Fonseca A., Nagy J.B., Moreau N., Delos M., Raymundo-Piñero E., Béguin F., Kirsch-Volders M., Fenoglio I. (2009). Structural defects play a major role in the acute lung toxicity of multiwall carbon nanotubes: Toxicological aspects. Chem. Res. Toxicol..

