# Supplementary material for: Prometastatic Potential of Non-Functionalized Multiwalled Carbon Nanotubes in the MDA-MB-436 Breast Cancer Cell Line Model
Source: Int J Mol Sci. 2025 Mar 19;26(6):2777. doi: 10.3390/ijms26062777 (PMC11943048; doi:10.3390/ijms26062777)

Supplementary Materials

Supplementary Table S1. Proteome Profiler Human Cytokine Array Kit list of detected proteins according to manufacturer's data

C5a	IL-1 beta	IL13	CXCL11/I-TAC
CD40 Ligand	IL-1 ra	IL-16	CCL2/MCP1
G-CSF	IL-2	IL-17	MIF
GM-CSF	IL-4	IL-17E	MIP-1 alpha/beta
CXCL1/GRO alpha	IL-5	IL-18	CCL5/RANTES
CCL1/I-309	IL-6	IL-21	CXCL12/SDF-1
ICAM-1	IL-8	IL-27	Serpin E1/PAI-1
IFN-gamma	IL-10	IL-3 alpha	TNF-alpha
IL-1 alpha	IL12 p70	CXCL10/IP-10	TREM-1

Supplementary Table S2. Proteome Profiler Human Apoptosis Array Kit list of detected proteins according to manufacturer's data

Bad	TRAIL R1/DR4	PON2
Bax	TRAIL R2/DR5	p21/CIP1/CDNK1A
Bcl-2	FADD	p27/Kip1
Bcl-x	Fas/TNFSF6	Phospho-p53 (S15)
Pro-Caspase-3	HIF-1 alpha	Phospho-p53 (S46)
Cleaved Caspase-3	HO-1/HMOX1/HSP32	Phospho-p53 (S392)
Catalase	HO-2/HMOX2	Phospho-Rad17 (S635)
cIAP-1	HSP27	Pro-Caspase-3
cIAP-2	HSP60	SMAC/Diablo
Claspin	HSP70	Survivin
Clusterin	HTRA2/Omi	TNF RI/TNFRSF1A
Cytochrome c	Livin	XIAP

Supplementary Table S3. MWCNTs parameters according to manufacturer's data

	Number of external walls	Outer diameter	Inner diameter	Length
5-20 nm MWCNTs	3-15	5-20 nm	2-6 nm	1-10 µm
50-80 nm MWCNTs	no data	50-80 nm	5-15 nm	10-20 µm

Supplementary Figure S1. Phalloidin-TRICT staining of MDA-MB-436 cells actin cytoskeleton after incubation with MWCNTs solutions for 48 hours. DAPI staining was performed to dye cells nuclei.

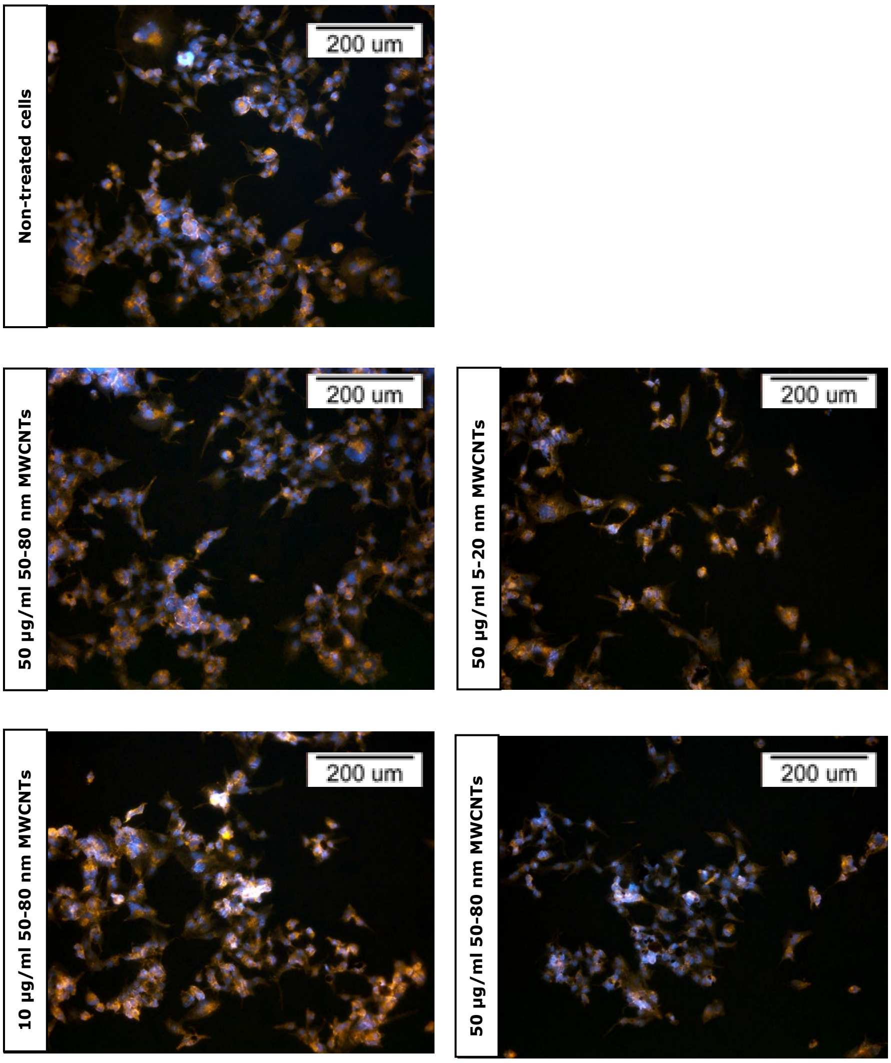

Supplement: Supplementary file 1 [file ijms-26-02777-s001.zip › ijms-3510683-supplementary.pdf]
